# A mixed-methods study exploring women’s perceptions of terminology surrounding fertility and menstrual regulation in Côte d’Ivoire and Nigeria

**DOI:** 10.1186/s12978-021-01306-5

**Published:** 2021-12-20

**Authors:** Grace Sheehy, Elizabeth Omoluabi, Funmilola M. OlaOlorun, Rosine Mosso, Fiacre Bazié, Caroline Moreau, Suzanne O. Bell

**Affiliations:** 1 Department of Population, Family and Reproductive Health, Johns Hopkins Bloomberg School of Public Health, 615 N. Wolfe St., Baltimore, MD 21205 USA; 2 Centre for Research, Evaluation Resources and Development, Ile-Ife, Nigeria; 3 Department of Community Medicine, University of Ibadan, Ibadan, Oyo Nigeria; 4 Statistics and Population Studies Department, University of the Western Cape, Cape Town, South Africa; 5 École Nationale Supérieure de Statistique et d’Économie Appliquée d’Abidjan (ENSEA), Abidjan, Côte d’Ivoire; 6 Institut Supérieur des Sciences de la Population, Université Joseph Ki-Zerbo, Ouagadougou, Burkina Faso

**Keywords:** Menstrual regulation, Period regulation, Pregnancy termination, Abortion, Reproductive health, Côte d’Ivoire, Nigeria

## Abstract

**Background:**

Women use various terms when discussing the management of their fertility and menstrual irregularities and may interpret the experience of ending a possible pregnancy in nuanced ways, especially when their pregnancy status is ambiguous. Our study aims to understand the terminology used to refer to abortion-like experiences (specifically menstrual regulation and pregnancy removal), and the specific scenarios that these practices encompass among women who reported doing something to bring back a late period or ending a pregnancy in Nigeria and Côte d’Ivoire.

**Methods:**

Our analysis draws upon surveys with women in Nigeria (n = 1114) and Cote d’Ivoire (n = 352). We also draw upon qualitative in-depth interviews with a subset of survey respondents in Anambra and Kaduna States in Nigeria, and Abidjan, Cote d’Ivoire (n = 30 in both countries). We examine survey and interview questions that explored women’s knowledge of terminology pertaining to ending a pregnancy or bringing back a late period. Survey data were analyzed descriptively and weighted, and interview data were analyzed using inductive thematic analysis.

**Results:**

We find that the majority (71% in Nigeria and 70% in Côte d’Ivoire) of women perceive menstrual regulation to be a distinct concept from pregnancy removal, yet there is considerable variability in whether specific scenarios are interpreted as referring to menstrual regulation or pregnancy removal. Menstrual regulation is generally considered to be more ambiguous and not dependent on pregnancy confirmation in comparison to pregnancy removal, which is consistently interpreted as voluntary termination of pregnancy.

**Conclusions:**

Overall, menstrual regulation and pregnancy removal are seen as distinct experiences in both settings. These findings have relevance for researchers aiming to document abortion incidence and experiences, and practitioners seeking to address women’s reproductive health needs.

**Supplementary Information:**

The online version contains supplementary material available at 10.1186/s12978-021-01306-5.

## Plain Language summary

Women use various words and phrases to describe their experiences managing their fertility and menstrual irregularities, and may interpret the experience of ending a possible pregnancy in nuanced ways, especially when their pregnancy status is ambiguous. Our study aims to understand the terminology women use to refer to abortion-like experiences (specifically menstrual regulation, which refers to actions taken to regulate a menstrual cycle, and pregnancy removal), and the specific scenarios that these practices encompass among women who reported having had an abortion in Côte d’Ivoire and Nigeria. Our analysis draws upon data from surveys and qualitative interviews conducted in both countries. We find that the majority (71% in Nigeria and 70% in Côte d’Ivoire) of women perceive menstrual regulation to be a distinct concept from pregnancy removal, yet there is considerable variability in whether specific scenarios are interpreted as referring to menstrual regulation or pregnancy removal. Menstrual regulation is generally considered to be more ambiguous and not dependent on pregnancy confirmation in comparison to pregnancy removal, which is consistently interpreted as voluntary termination of pregnancy. These findings have relevance for researchers aiming to document abortion incidence and experiences, and practitioners seeking to address women’s reproductive health needs.

**Supplementary Information:**

The online version contains supplementary material available at 10.1186/s12978-021-01306-5.

## Introduction

Achieving the Sustainable Development Goals (SDGs) requires addressing long neglected sexual and reproductive health issues, such as access to safe abortion [1]. Unsafe abortion remains a leading contributor to maternal mortality and morbidity in many parts of the world, particularly those where access to safe abortion care is restricted. The liberalization of abortion laws and policies has been associated with reduced rates of unsafe abortion and related complications in a number of settings, including Bangladesh, Romania and South Africa [2, 3]. In some settings where access to comprehensive abortion care remains legally restricted, a range of efforts have been undertaken by communities, organizations and governments to reduce the harms associated with unsafe abortion and increase access to methods for the early termination of pregnancy*.* Menstrual (or period) regulation refers to actions taken to regulate the menstrual cycle, i.e. to bring on a late period [4]. Women may interpret the experience of ending a possible pregnancy in nuanced ways, especially in circumstances when one’s pregnancy status is ambiguous [5] or specifically in the absence of or prior to a pregnancy test being taken, when one’s pregnancy status is unconfirmed clinically. In the first trimester, a pregnancy loss (affecting up to 20% of all pregnancies) may be experienced as a delayed period as women may not be aware of the pregnancy [5, 6]. Irregular menstrual cycles can also delay the identification of a pregnancy; this may be particularly relevant in low-resource settings, where malnutrition may contribute to menstrual irregularity [7, 8]. For some women, deciding not to confirm a pregnancy can be advantageous to their sense of self and their social reputation—Bell and Fissell describe this state of uncertainty surrounding pregnancy identification as “productive ambiguity” [5]. This ambiguity can allow women to seek treatment without identifying the treatment as an abortion due to the absence of pregnancy confirmation. For instance, in a qualitative study in Latin America, respondents perceived their medical abortion as being akin to regulating a period, stating they did not consider it to be an abortion since they were early in pregnancy [9]. Even recent research in the United States suggests a demand for menstrual regulation (specifically “missed period pills”), which some women interpreted as an alternative to abortion that would allow them to avoid knowing if they were pregnant, providing “less moral conflict” [10].

Research demonstrates that women in a range of settings, including countries in Africa, Asia and the Caribbean, use various turns of phrase to refer to the experiences of managing their fertility, including bringing back a late period or ending a pregnancy. The practice of menstrual regulation is well-known and codified into family planning programs and policy in Bangladesh [11]. While abortion is legally restricted except to save a woman’s life in Bangladesh, menstrual regulation has been available as “an interim method for establishing non-pregnancy” since 1974 [11, 12]. Menstrual regulation is allowed if a pregnancy has not been confirmed and a woman’s last period was up to 10–12 weeks ago, depending on the type of provider, and up to 9 weeks ago when using a medication regimen (mifepristone and misoprostol) [11, 12]. The provision of menstrual regulation services in this setting has been linked to a decline in maternal mortality, with 78.3% reduction in abortion-related mortality observed in government-served parts of rural Matlab [13].

Although there is a dearth of literature on menstrual regulation outside of Bangladesh, the available evidence suggests this phenomenon may be relevant in other parts of the world. For instance, research in Malaysia found that women use terms like “cleanse”, “discard”, and “wash” to describe ending a pregnancy [14], while in the Caribbean, women ask providers to “bring on their period” [15]. The phenomenon of bringing back a late period has also been explored in some African countries, including Nigeria, one of the settings of our study. For instance, Renne [16] described how women in southwestern Nigeria use various medicines and procedures to “clean the inside” (i.e. the womb), in order to bring back menstruation. Ethnographic research among young women in rural Tanzania documented a practice referred to as “suspending a pregnancy” or “moving a pregnancy to the back”, such that a pregnancy was delayed using traditional medicines until a young girl or woman is ready to be pregnant; the practice was not considered to carry the stigma of an abortion [17]. In recent population-based survey research on induced abortion in Côte d’Ivoire and Nigeria, researchers found substantial levels of self-reported menstrual regulation (20.8 per 1000 in Côte d’Ivoire and 28.3 per 1000 in Nigeria) [18]. While there was overlap in the methods used for menstrual regulation and those used for pregnancy termination, menstrual regulations more often involved the use of pills and traditional methods, and interaction with non-clinical providers.

The ways women refer to, and understand terminology pertaining to, their reproductive health has implications for researchers, clinicians, and public health practitioners. In order to better address the full spectrum of women’s sexual and reproductive health needs, understand their reproductive health behaviors and preferences, and accurately capture these experiences through survey research, we need to improve our understanding of women’s knowledge and use of concepts relating to regulating a period and ending a pregnancy. There remains a gap in the literature regarding how women understand the concepts of pregnancy termination and menstrual regulation, particularly in sub-Saharan Africa. This topic may be particularly salient in settings where most abortions are criminalized, where masking or obscuring a procedure for dealing with an unwanted pregnancy may be especially advantageous to a woman’s social safety. Our study focuses on two such countries: Côte d’Ivoire and Nigeria. In Côte d’Ivoire, abortion is legal only in cases of rape or to save a woman’s life, and in Nigeria, only to save a woman’s life. In both Côte d’Ivoire and Nigeria, most abortions are unsafe and procured in the informal sector; in 2018 nearly two-thirds of abortions in both countries involved the use of risky, non-recommended methods provided by non-clinical sources [19]. Specifically, in Nigeria, 50.2% of abortions in 2018 were provided by a traditional or other non-clinical provider, and 12% were provided by a chemist or pharmacist, while in Côte d’Ivoire, 56.8% of abortions were provided by a traditional or other non-clinical provider and 2.7% by a chemist or pharmacist [20, 21]. Rates of maternal mortality are high in both countries, with estimates ranging from 502 to 944 deaths per 100,000 live births in Côte d’Ivoire and 496 to 814 per 100,000 live births in Nigeria [22–24]; in both settings, unsafe abortion is considered a significant contributor [22, 25]. While both countries have seen reductions in their maternal mortality rates since the creation of the Millennium Development Goals, neither is currently on track to achieve the reduction in rates aimed for in SDG 3 [26]. Achieving the SDGs will require improving the provision of comprehensive abortion care (including safe abortion care, post-abortion care and contraceptive counseling) [1, 27]. Further, ensuring women have access to reproductive health care in a way that is contextually resonant and rooted in their desires, needs and priorities is imperative for attaining reproductive justice*.*

Using a mixed-methods approach, our study aims to (1) Explore Ivorian and Nigerian women’s understanding of the terms “menstrual (or period) regulation when worried she might be pregnant” and “pregnancy removal”; and (2) Understand how perceptions of “menstrual/period regulation” and “pregnancy removal” vary by women’s sociodemographic characteristics and the circumstances surrounding the event (e.g. whether or not a pregnancy has been confirmed).

## Data and methods

### Study design

This analysis draws upon data from two quantitative surveys and in-depth qualitative interviews conducted by Performance Monitoring for Action (PMA) in Nigeria with its implementing partner, the Centre for Research, Evaluation Resources and Development (CRERD), and in Côte d’Ivoire with l’Institut National de la Statistique de Côte d’Ivoire (INS-Côte d’Ivoire) and la Direction de la Coordination du Programme National de Santé de la Mère et de l’Enfant (DC-PNSME) (initial survey and qualitative), and the École Nationale Supérieure de Statistique et d'Economie Appliquée of Abidjan (ENSEA) (follow-up survey). The Nigeria survey, conducted in April–May 2018, used a three-stage cluster sampling design. Seven states were selected using probability proportional to size (PPS) sampling. In each state, geographic clusters of approximately 200 households were selected using PPS, and 35 (40 in Lagos) households were randomly selected per cluster. The Côte d’Ivoire survey, conducted in July–August 2018, used a two-stage cluster sampling design. Seventy-three geographic clusters were selected using PPS, and 35 households were randomly selected per cluster. Data were collected by women recruited from within or near the geographic enumeration areas and trained in data collection techniques [28]. Each data collector was assigned a random selection of households. All women ages 15 to 49 who normally reside or slept the night before in the sampled households were invited to participate in the female survey; those who were incapable of participating due to language or physical or mental health issues were excluded. The final sample included 11,106 women in Nigeria and 2738 women in Côte d’Ivoire (both a 98.1% response rate).

Interviews began with the interviewer introducing themselves and the PMA project, explaining the purpose of the survey, and obtaining consent from respondents. The surveys included a module on abortion which posed a range of questions on women’s abortion knowledge and experiences. Prior to asking questions about abortion, interviewers informed respondents of the topic, noted that abortion is a common experience and explained that this study simply aims to understand it better. They also reminded respondents that the survey is confidential and that any question can be skipped. We incorporated several measures to improve the quality of self-reported abortion data, including different terminology to refer to a possible abortion: women were asked separately whether they had ever done something to “remove a pregnancy” or to “bring back a period when they were worried they might be pregnant.” We classified both of these events as a potential abortion. These phrases emerged from the pilot, during which we discussed with interviewers the phrases and terminology women use to discuss actions taken that could be interpreted as abortion. We included these phrases in pilot interviews prior to data collection and they were overwhelmingly interpreted correctly by participants [29].

We conducted a follow-up survey in both countries (excluding Kano state in Nigeria) from November 2019–February 2020 (Nigeria) and October–November 2020 (Côte d’Ivoire) with respondents who reported having done something to remove a pregnancy or bring back a period in the 2018 survey, and who consented to be re-contacted (n = 1452 in Nigeria and n = 426 in Côte d’Ivoire). Interviews were conducted with 1144 women in Nigeria (78% response rate) and 358 in CDI (84% response rate). Some respondents were excluded from the follow-up survey because they had participated in pilot testing or qualitative interviews, and some for logistical reasons. Others were lost to follow-up because they had moved, were unreachable, declined to participate or had passed away. The aim of the follow-up survey was to explore women’s abortion experiences in greater detail. We used this opportunity to ask about their knowledge and perceptions of the two abortion-related terminologies used in the initial 2018 survey (menstrual regulation and pregnancy removal). All respondents provided informed consent to participate and surveys were administered face-to-face in a private location convenient to the respondent. For this paper, we limited our analytic sample to women who completed the follow-up survey and had complete data on our key measures of interest, particularly questions pertaining to terminology (n = 1114 in Nigeria and n = 352 in Côte d’Ivoire).

In addition, a sub-set of women from both countries who reported having had an abortion in the original survey were invited to speak about their experience in more depth in a qualitative interview in late 2018. All women who reported doing something to remove a pregnancy or bring back a late period in the initial survey were asked whether they would be interested in participating in a follow-up in-depth interview. Among women who agreed to be contacted for a follow-up interview, we purposively selected women residing in Abidjan (in Côte d’Ivoire), and in Anambra and Kaduna states (in Nigeria) for feasibility. We aimed to include women who had used medication abortion when possible: in Nigeria we purposively sampled women who received abortion pills from a pharmacy or who received pills or a surgical procedure from a clinical source, while in Côte d’Ivoire we sampled women who had received abortion pills or a surgical procedure from any source. We conducted 30 in-person, in-depth interviews in each country, for a total of 60 interviews. Women provided verbal informed consent to participate, and qualitative interviews were audio-recorded with participant consent. During the consent process, participants were reminded that their participation is voluntary, and that they could skip a question, pause or stop the interview at any time. Interviews were conducted in locations with auditory privacy, and visual privacy when possible. All data collectors received training on the ethical conduct of research, and qualitative interviewers received additional training on conducting research on topics which may be sensitive, such as abortion. If a respondent showed signs of distress during the interview, interviewers offered to stop the interview and offered resources for additional support. Interviews followed a semi-structured interview guide and were conducted in French in Côte d’Ivoire, and in Igbo, Hausa and English in Nigeria. All interviews were transcribed verbatim; the Nigerian interviews were translated into English where necessary while the Cote d’Ivoire transcripts were analyzed in French. All interview recordings and transcripts were uploaded to a secure server. This study received ethics approval from the Johns Hopkins Bloomberg School of Public Health, the National Health Research Ethics Committee of Nigeria, and the Comité National d'Étique de la Recherche (CNER) in Côte d'Ivoire.

### Measures

Our quantitative outcomes of interest were participants’ knowledge and interpretation of two abortion-related terminologies: pregnancy removal and menstrual regulation. In the follow-up survey, we included several questions to explore respondents’ understanding of these concepts. Respondents were asked whether they viewed pregnancy removal and menstrual regulation as the same or different experiences; response options included same, different, and don’t know, and those who responded “don’t know” were grouped with “different” in a binary variable. We performed a sensitivity analysis excluding “don’t know” and the results did not change significantly. Respondents were then presented with nine scenarios and asked whether each scenario referred to removing a pregnancy (yes/no) and/or regulating a period when a woman is worried she is pregnant (yes/no). The scenarios included:

- Taking a pill within a couple days after unprotected sex;
- Taking pills after missing one or two periods without pregnancy confirmation;
- Having a procedure after missing one or two periods without pregnancy confirmation;
- Taking pills when a woman is sure she is early in a pregnancy;
- Having a surgery when a woman is sure she is early in a pregnancy;
- Taking pills when the pregnancy has been confirmed;
- Having a surgery when the pregnancy has been confirmed;
- Taking pills after a miscarriage; and
- Having a surgery after a miscarriage

We considered a range of sociodemographic characteristics as explanatory variables in the quantitative analysis, including age, marital status, residence (urban, rural), education, state (Nigeria), parity, ethnicity, and religion. We also explored abortion-specific measures such as how certain the respondent was that she was pregnant when she had a pregnancy removal or menstrual regulation (very certain/certain, not certain), whether she took a pregnancy test to confirm the pregnancy that was terminated, the method she used to terminate her pregnancy (surgery, medication abortion, other pills, traditional/other methods), and the terminology she used to describe the event (pregnancy removal, menstrual regulation).

In the qualitative interviews, we asked several questions to explore women’s knowledge and understanding of various terminology relating to a potential pregnancy termination. We first asked participants an open-ended question: “What are the different ways that you have heard women refer to this experience of ending a pregnancy?” We then followed-up with a series of probes to explore their understanding of the phrases “removing a pregnancy” and “regulating a period when worried about pregnancy,” and what they saw as similarities and/or differences in these two concepts.

### Data analysis

We used descriptive statistics to examine the distribution of the variables of interest. We assessed bivariate distributions of the outcome variables (pregnancy removal and period regulation) according to respondent background characteristics and abortion-specific measures. We then examined the nine scenarios according to whether respondents perceived the two concepts to be different, overall and by pregnancy certainty and whether they reported a menstrual regulation or pregnancy removal in the initial survey. To assess whether differences were statistically significant we used design-based F statistics and paired and unpaired t-tests. Data were weighted to account for the complex sampling design and clustering. We analyzed all survey data using Stata 16.1.

We analyzed interview data for content and themes using both deductive and inductive approaches. The lead author (GS) and a second researcher first read through the transcripts and wrote summaries of each interview. We then developed a codebook based on the interview guide and research questions. We uploaded transcripts to Atlas.ti 8 for coding. As we coded interviews, we supplemented the codebook with inductive codes that emerged from the data. We used codes to organize the text according to our a priori domains of inquiry around terminology (including perceptions of pregnancy removal and period regulation, and differences and similarities between them), while in vivo codes helped us document new turns of phrase supplied by participants. We used coded output as well as full interview transcripts to explore women’s understanding and use of terminology within and across interviews*.* We wrote analytic memos during the coding process to guide our interpretation and identification of early themes. Following the completion of coding we conducted an inductive thematic analysis, whereby we developed broader categories based on the initial codes and quotes from the interviews, eventually grouping these into several key themes.

## Results

### Sample characteristics

The mean age of respondents was 31.9 in Nigeria and 31.8 in Côte d’Ivoire (Additional file 1: Table S1). In Nigeria, most respondents had a secondary education (50.5%) or higher (26.0%), and were currently married or cohabiting (71.4%). Most identified as Catholic (17.5%) or other Christian (52.8%), while roughly one-quarter (27.6%) identified as Muslim. Three-quarters already had children, and almost two-thirds (61.0%) resided in urban areas. In Côte d’Ivoire, nearly one-third (32.1%) had never attended school, while 37.5% had a primary education. Most (65.1%) were married or cohabiting, nearly 90% had children, and nearly two-thirds (63.1%) resided in urban areas. In our qualitative samples, the average ages were 30.5 in Nigeria and 29.4 in Côte d’Ivoire. In both countries, most qualitative participants had a secondary education or higher, were married or cohabiting, and already had children.

### Survey results

#### Perceptions of terminology by characteristics

In both Nigeria and Côte d’Ivoire, more than two-thirds of women considered period regulation and pregnancy removal to be different (70.8% and 69.9% respectively); this did not vary significantly according to any social or demographic characteristics (Additional file 2: Table S2). However, women who were less certain that they had been pregnant when they took steps to end their pregnancy (or suspected pregnancy) were significantly more likely to consider period regulation and pregnancy removal to be different: in Nigeria, 85.5% of women who were not certain considered the two concepts to be different, compared to 66.5% of women who were very certain (p < 0.001), and in Côte d’Ivoire, 89.4% who were not certain considered the two concepts to be different compared to 65.7% who were very certain (p = 0.02). In Nigeria, women who reported having regulated a period were significantly more likely than those who reported a pregnancy removal to consider these concepts to be different (75.8% versus 68.4%, p = 0.01).

### Perceptions of specific period regulation and pregnancy removal scenarios

Respondent perceptions of which terms (pregnancy removal and period regulation) applied to each scenario varied considerably, and there were significant differences for each of the nine scenarios (Additional file 3: Table S3). In scenarios where a pregnancy was confirmed, or where surgery was involved, far more women considered these to be a pregnancy removal than a period regulation. The majority of women in both countries considered having a surgery once a pregnancy was confirmed to be pregnancy removal (79.7% in Nigeria, 67.1% in Côte d’Ivoire) whereas a much smaller percentage (12.0% in Nigeria, 16.8% in Côte d’Ivoire) considered this to be period regulation. In scenarios where a pregnancy was *not* confirmed and pills were used, more women considered these situations to refer to period regulation than pregnancy removal. For instance, in a scenario in which a woman had missed one to two periods and taken pills without pregnancy confirmation, 56.5% and 65.1% considered this to be a period regulation and 45.4% and 33.5% considered it to be a pregnancy removal in Nigeria and Côte d’Ivoire, respectively. Some differences were noted between the two countries: in Côte d’Ivoire, women were more likely to agree that scenarios referred to period regulation than to pregnancy removal. For instance, considerably more women agreed that taking pills or having a surgical procedure without pregnancy confirmation was considered a period regulation in Côte d’Ivoire (65.1% and 58.0%) than in Nigeria (56.5% and 26.1%).

### Perceptions of scenarios according to whether women reported period regulation or pregnancy removal

Across both countries, there were significant differences in whether respondents considered certain scenarios to refer to a period regulation or a pregnancy removal based on how they reported their own experience of ending a pregnancy (Additional file 4: Table S4). In particular, those who reported removing a pregnancy viewed more scenarios as pertaining to both period regulation and pregnancy removal than did women who reported regulating a period.

In both countries women who reported removing a pregnancy were significantly more likely to agree that scenarios without pregnancy confirmation pertained to a pregnancy removal than women who reported regulating a period (p < 0.05). In Côte d’Ivoire, women who reported a pregnancy removal perceived more scenarios to refer to a pregnancy removal than did women who reported regulating a period. In Côte d’Ivoire there were also lower levels of agreement that scenarios without pregnancy confirmation referred to pregnancy removals than in Nigeria, however similar to Nigeria, women who reported removing a pregnancy were significantly more likely to consider these to be pregnancy removals than women who reported regulating a period. Women who reported regulating a period in Côte d’Ivoire had the lowest levels of agreement that any scenario pertained to pregnancy removal.

### Perceptions of scenarios according to own certainty about pregnancy that was terminated

Finally, we examined the nine scenarios according to whether respondents were certain or uncertain they were pregnant at the time they had their potential abortion (Additional file 5: Table S5). In both countries, women who were certain they were pregnant were significantly more likely to perceive scenarios where a woman was sure she was pregnant as referring to period regulation than women who were not certain about their pregnancy. In Côte d’Ivoire, women who were certain about their pregnancy were also more likely to consider taking a pill a couple days after sex as a period regulation than women who were not certain (56.5% vs. 42.4%, p = 0.04). In terms of pregnancy removal scenarios, in Nigeria, more women who were certain they were pregnant perceived taking pills prior to pregnancy confirmation to refer to a pregnancy removal than women who were not certain (46.4% vs. 37.8%, p = 0.04). In Côte d’Ivoire, more women who were certain about their pregnancy perceived having a surgical procedure either with or without pregnancy confirmation to refer to a pregnancy removal than women who were not certain about their pregnancy (p < 0.05).

### Interview findings

#### Pregnancy removal was seen as synonymous with abortion

Participants in Abidjan and both Nigerian states had a clear understanding of what an abortion was, although some preferred to use terminology that did not explicitly mention abortion, including but not limited to “removing a pregnancy.” In Abidjan, many women were comfortable using the word “abortion” and also referred to “curettage” as a commonly used term for abortion. They were also familiar with a range of other phrases including “washing the stomach”, “skipping”, “dropping”, “spoiling a pregnancy”, and “getting rid of the pregnancy”. Some of the language used by women in Nigeria was similar to that of women in Abidjan; for instance, one 25-year old participant in Kaduna described how some women talk about going to the hospital “to wash their womb.” Additional file 6: Fig. S1 presents word clouds highlighting the words women used to refer to the experience of ending a pregnancy in Côte d’Ivoire (S1a) and Nigeria (S1b), all translated into English.

We probed women’s knowledge of the concept of “pregnancy removal/removing a pregnancy” in more depth. Nearly all participants in both countries were familiar with this phrasing and perceptions of “removing a pregnancy” were similar across both settings: it was widely seen as synonymous with abortion, i.e. an intentional act of terminating a pregnancy, even among women who were not previously familiar with this specific phrase. As one 24-year old respondent in Kaduna said: “Abortion is removing a pregnancy so no one knows about it.” In Abidjan, women referred to removing and ending a pregnancy interchangeably. Many women in both countries consistently referred to their own abortion experiences as “removing a pregnancy” throughout their interviews.

### Understandings of menstrual regulation were more varied

In contrast to their common understanding of pregnancy removal and abortion, perceptions of menstrual regulation varied considerably among participants in both countries. Several participants in each setting were unfamiliar with this behavior, while those who were familiar largely described it as doing something to bring back a late period. Opinions regarding whether menstrual regulation and pregnancy removal referred to the same or different acts were divided. Some participants said there was no difference between the two concepts and believed they both referred to terminating a pregnancy, in that the intention of both acts was to re-establish menstruation. Particularly in Abidjan among women who saw the two as the same, use of the phrasing “bringing back a period” could be strategic in order to reduce the stigma associated with an abortion. For others, menstrual regulation could mean avoiding a pregnancy that had not yet been established: one 40-year old participant in Abidjan stated “To me it means, in order to not become pregnant, she will drink a medication so that her period comes.” In Abidjan, a few participants described how distinguishing between the concepts could be a matter of framing the issue in order to get access to treatment:

*How to bring [a period] back? We go to see the women [drug sellers] and we have to lie to them. Ah, my period doesn’t come, that’s it, so she’ll give us very strong drugs to make, if you’re pregnant, to make the pregnancy go away, or [if] the menstrual cycle hasn’t arrive yet, we rush it—*28-year old woman in a relationship with no children who works as a secretary in Abidjan

According to some participants, whether or not a woman was seeking to remove a pregnancy or regulate a period also had bearing on the methods she used—pregnancy removal was associated with more surgical methods, while menstrual regulation was associated with pills or traditional remedies:

*Removing a pregnancy, that’s doing curettage, like you don’t want the child anymore. You want to remove it … but if you’re going to do something to get your period back, this is a pill—it’s not the same thing. The one, she wants to end the pregnancy … The other, she has doubts, so she wants to take the pill so that … her period can come quickly—*27-year old, engaged woman with two children who works as an esthetician in Abidjan

Regarding perceptions of terminology according to participant characteristics, in Abidjan the majority of young women with no children said there was no difference between menstrual regulation and pregnancy removal (n = 5 out of 7 nulliparous women under the age of 25). Most women in our sample had a secondary school education or higher (25 out of 30 in Nigeria and 23 out of 30 in Côte d’Ivoire), and thus we were limited in our ability to compare patterns in responses according to education level. Women of all education levels were familiar with a range of terms for ending a pregnancy and had heard of menstrual regulation. In Abidjan, most women with a primary education or less considered menstrual regulation and pregnancy removal to be the same thing, while there was more variability among women with higher levels of education.

### Menstrual regulation and pregnancy removal were distinguished by several factors

We identified three factors in our interviews as particularly relevant to women’s understanding of menstrual regulation and whether it was distinct from pregnancy removal, which represent a continuum of pregnancy certainty: the possibility of being pregnant, whether a pregnancy had been confirmed, and how early a confirmed pregnancy was.

In Abidjan, many women distinguished between menstrual regulation and pregnancy removal based on the *likelihood* of being pregnant, specifically whether or not a woman had engaged in sexual relations during the time of the month when she was most likely to be fertile. For these women, the terminology used to describe the experience was less important than the probability of being pregnant, which determined whether or not an experience was considered an abortion. As one participant described:

*You know in your soul and your conscience whether you had sexual relations. Between us, as women, we know ... If you know that you had relations during your fertile time … and you say you want to bring back your period, it’s the same thing as ending a pregnancy because you know… your period is late, and a late period is a pregnancy—*28-year old, single woman with one child who works as a shopkeeper in Abidjan

Moving beyond the *probability* of pregnancy to the *confirmation* of pregnancy, we found in both countries that whether or not a pregnancy had been confirmed was often a primary factor distinguishing menstrual regulation and pregnancy removal. There was a clear sense that a woman removes a pregnancy when she *knows* she is pregnant, and regulates a period when she *could be* pregnant:

*They [menstrual regulation and pregnancy removal] are two different things…Because when a pregnancy is removed, it is regarded as abortion, but when a woman misses her period it might not necessarily be pregnancy—*25 year old, divorced woman with no children from Kaduna State

Pregnancy confirmation did not always involve clinical confirmation—several participants described how there are distinct signs in a woman’s body that let her know she is pregnant. Thus, in the absence of those signs one might be more likely to believe they were regulating a period than removing a pregnancy. Menstrual stoppage was considered a key sign of pregnancy, although often after some time had passed. Some participants distinguished between menstrual regulation and pregnancy removal based either on how late a woman’s period was or how early in pregnancy she was:

*So we don’t see this as a pregnancy that is being ended when the person has a period that’s 2 weeks late, 1 month late, and she has to go to the hospital and take medication so her period comes back … we don’t see that as a pregnancy—*42-year old, married woman with five children who works as a teacher in Abidjan

### Menstrual regularity was valued

For many women, menstrual stoppage was not inherently tied to pregnancy, thus taking steps to bring on a period was not necessarily akin to having an abortion. In particular, several women mentioned that a woman’s period could cease or be delayed if she had an illness, due to antibiotics, or when she is breastfeeding. As such, while taking pills to bring on a period was seen as potentially relating to pregnancy, it was also considered at times to be more of an effort to improve a woman’s health or as a marker of fertility:

*Some women may not have their menstrual flow at the time it is supposed to come, so they take pills for it to resume […]. If you resume menstruation, you will feel healthy—*48-year old, married woman with six children from Kaduna State

*In my understanding, when the menstrual flow begins after childbirth, it signals that she can get pregnant at any time … it means the body is back to work—*30 year old, married woman with two children from Kaduna State

Further highlighting the value of menstrual regularity, several women described how they paid close attention to their menstrual cycle and its consistency and flow month-to-month. In Abidjan, one participant described how she regularly took medicines to regulate her period, specifically with the aim of improving the flow of her menses:

*My period came, but not how I wanted it to. It came a little and then it stopped, so I wanted a medicine that can make it come well. When I explain the problem to [the drug vendor], she gives me the medicine, and I wait for [my period] to come—*27-year old single woman with one child who works as a shopkeeper in Abidjan

## Discussion

Most of our respondents considered menstrual regulation and pregnancy removal to be distinct experiences. However, when considered in more nuance (i.e. when survey respondents were presented with specific scenarios and when qualitative participants were probed further) there was considerable variability in women’s perceptions of the two concepts. Importantly, both our qualitative and quantitative findings showed that whether a pregnancy had been confirmed was a key marker of whether an act was considered menstrual regulation or pregnancy removal. Our qualitative findings confirmed that in the absence of a pregnancy test or unequivocal pregnancy symptoms, women were open to the possibility that actions could be taken to bring back a period which could be late for myriad reasons.

In qualitative research in Latin America, women described use of medication abortion early in pregnancy as a process distinct from an induced abortion; this framing helped them accept their decision to end a pregnancy [9]. As one participant from Peru explained: “What I did was regulate my period. I’m not going to accept that I’ve had an abortion … because I was barely a month pregnant.” Similarly, women in the United States described how a lack of pregnancy confirmation would provide them with a “psychological cushion” and be better for their emotional well-being if they were to take a “missed period pill” [10]. Thus, similar to women in our study, women in these settings viewed menstrual regulation and pregnancy removal as distinct experiences, and being *early in pregnancy* in particular helped them frame menstrual regulation as distinct from an abortion. Although this framing may allow women to distance themselves from the stigma associated with abortion, women in Dhaka, Bangladesh described in qualitative interviews how menstrual regulation still has strong stigma attached to it in that context [30]. Similar to the views of some of our participants who did not consider menstrual regulation and pregnancy removal to differ, in Bangladesh menstrual regulation was still believed to carry shame much like induced abortion.

The distinct framing of women’s experiences according to the stage of pregnancy may also reflect different perspectives between a provider’s clinical assessment of pregnancy onset and women’s own recognition of pregnancy. Our qualitative participants described how having recently missed a period did not necessarily mean a woman was pregnant. It is possible that for some women, menstrual regulation and pregnancy removal exist on a continuum, and the actions they take to regulate their fertility depend on how certain they are that they are pregnant, irrespective of whether a pregnancy has been confirmed clinically.

The meaning of menstrual regulation extended beyond the notion of pregnancy as participants also spoke of the importance of menstrual regularity in signifying good health and fertility. These findings are similar to research in numerous settings which has documented how women often perceive menstruation as getting rid of “bad blood” and signifying good health [31, 32]. For instance, in northern Ethiopia, women described the important biological function of menstruation in demonstrating that “the body is ready to reproduce” [33]. These thoughts were echoed by our participants, who considered the return of menstruation to mean “the body is back to work.” Thus, the motivation to return a late menses cannot only be interpreted as a potential early termination; for some women these actions are taken out of concern for their health and fertility.

Designing research instruments based on the language used and understood by women locally is imperative for generating accurate and precise estimates of health indicators. Recent population-based research on abortion incidence in Côte d’Ivoire, India and Nigeria included questions asking women about their experiences with both pregnancy removal and period regulation [19–21, 34]. A considerable number of women reported having experienced a period regulation but not a pregnancy removal. Including these events as pregnancy terminations would increase abortion incidence estimates by 24% to more than 100%; the researchers included half of reported menstrual regulations in their final abortion incidence calculations to account for the range of circumstances described as menstrual regulation, which do not all correspond to pregnancy termination as illustrated in this study [5]. While we encourage including questions about menstrual/period regulation to capture a wider spectrum of women’s actions to regulate their fertility, we also stress the importance of including clarifying questions regarding the motivation behind bringing back one’s period, one’s pregnancy certainty (including pregnancy test), and the number of periods missed to help distinguish period regulations for health reasons from those for fertility-regulation reasons. Further, understanding the language women use to refer to these experiences could be valuable in informing advocacy efforts to expand access to and use of safer abortion methods outside the formal health care system.

Regardless of the terminology women use to refer to dealing with an unwanted pregnancy, too few women have access to safe, effective abortion methods. Although not codified into family planning policy outside of Bangladesh, menstrual regulation has relevance to women’s reproductive health in numerous countries, particularly in settings where abortion is legally restricted, including Côte d’Ivoire and Nigeria. Framing these experiences as menstrual regulation could be beneficial for women’s sense of self and social reputation and expansion of access to menstrual regulation methods, including medication abortion drugs, could reduce complications from unsafe abortion. Further research is needed on women’s perceptions and experiences of menstrual regulation to inform these efforts in a wider range of countries. In addition, women’s ability to make autonomous decisions about their fertility and reproductive health leads to improved reproductive health outcomes [35, 36]. In order to exercise their reproductive choices, and to improve reproductive health outcomes in line with the SDGs, access to comprehensive reproductive health care is imperative and should include services for early termination of pregnancy or suspected pregnancy. Further, training for health professionals should address anti-abortion stigma through values clarification exercises and encourage non-judgmental conversations with patients about abortion and unwanted pregnancy. Efforts to expand access to safe abortion within existing legal frameworks in the short-term, and reforming restrictive abortion laws in the long-term, are also warranted.

Our study is not without limitations. Our analytic sample includes only women who reported removing a pregnancy or regulating a period and thus our findings are not representative of Ivorian or Nigerian women’s views on these phenomena more broadly. To the extent that women’s views on these concepts differ by whether they report a likely-abortion could impact the observed relationships. However, we do not suspect this potential bias would qualitatively change our conclusions with regard to these concepts being distinct and the role of pregnancy confirmation and method in distinguishing them. In addition, in our original quantitative survey we asked questions about “bringing back a late period” while the follow-up survey asked questions specifically about menstrual regulation, which may have been less well understood by respondents.

Our study also has a number of strengths. It is one of few that sheds light on the understudied phenomenon of menstrual regulation in a sub-Saharan African context and is the first to use both qualitative and quantitative methods to examine abortion-related terminology. Our data involve large and diverse samples of women who have had a range of abortion experiences, including those that occurred in clinic and non-clinical settings. Our data also included a number of factors that could be examined in association with our outcomes of interest, including certainty of pregnancy, whether they took a pregnancy test, and how they reported their potential abortion.

## Conclusions

The majority of women in our study stated that menstrual regulation and pregnancy removal were different. However, there was significant variability in the specific scenarios that women interpreted as being a pregnancy removal or menstrual regulation, and menstrual regulation was generally considered to be more ambiguous and not dependent on pregnancy confirmation. These findings expand our understanding of how women think and talk about pregnancy termination along a spectrum, and has implications for research on measurement, policy and practice that explores avenues to support safer strategies for women to control their fertility.

## Supplementary Information

**Additional file 1: Table S1.** Quantitative and qualitative sample characteristics.

**Additional file 2: Table S2.** Percentage that consider pregnancy removal different than period regulation, by background characteristics.

**Additional file 3: Table S3.** Perceptions of scenarios.

**Additional file 4: Table S4.** Perceptions of scenarios according to whether respondent reported menstrual regulation or pregnancy removal.

**Additional file 5: Table S5.** Perceptions of scenarios according to whether respondent was certain or not about being pregnant for the pregnancy that was terminated.

**Additional file 6: Fig. S1.** Word clouds highlighting terminology used by qualitative interview participants in Cote d’Ivoire and Nigeria.

## Data Availability

The datasets analysed during the current study are available in a public, open access Performance Monitoring for Action (PMA) repository [PMAData.org].

## Abbreviations

CNER: Comité National d'Étique de la Recherche
CRERD: Centre for Research, Evaluation Resources and Development
DC-PNSME: Direction de la Coordination du Programme National de Santé de la Mère et de l’Enfant
ENSEA: École Nationale Supérieure de Statistique et d'Économie Appliquée of Abidjan
INS-Côte d’Ivoire: L’Institut National de la Statistique de Côte d’Ivoire
PMA: Performance Monitoring for Action
PPS: Probability proportional to size
SDG: Sustainable Development Goals
