# Supplementary material for: A mixed-methods study exploring women’s perceptions of terminology surrounding fertility and menstrual regulation in Côte d’Ivoire and Nigeria
Source: Reprod Health. 2021 Dec 20;18:251. doi: 10.1186/s12978-021-01306-5 (PMC8686364; doi:10.1186/s12978-021-01306-5)
Supplement: Supplementary file 1 — Additional file 1: Table S1. Quantitative and qualitative sample characteristics. [file 12978_2021_1306_MOESM1_ESM.docx]

| **Table S1. Quantitative and qualitative sample characteristics (quantitative % weighted, N unweighted)** | | | | | | | | | | |
| --- | --- | --- | --- | --- | --- | --- | --- | --- | --- | --- |
|  |  | **Nigeria** | | | | | **Cote d'Ivoire** | | | |
|  |  | **Quantitative** | | **Qualitative** | | **Quantitative** | | | **Qualitative** | |
|  |  | % | N | % | N | % | | N | % | N |
| Total |  |  | 1114 |  | 30 |  | | 352 |  | 30 |
| Mean age | |  | 31.9 |  | 30.5 |  | | 31.8 |  | 29.4 |
| **Age** | |  |  |  |  |  | |  |  |  |
|  | 15-19 | 5.0 | 56 | 6.7 | 2 | 5.7 | | 20 | 10.00 | 3 |
|  | 20-24 | 15.0 | 167 | 20.0 | 6 | 16.8 | | 59 | 20.00 | 6 |
|  | 25-29 | 21.3 | 237 | 33.3 | 10 | 20.5 | | 72 | 26.70 | 8 |
|  | 30-34 | 20.7 | 230 | 10.0 | 3 | 18.8 | | 66 | 13.30 | 4 |
|  | 35-39 | 17.3 | 193 | 3.3 | 1 | 20.2 | | 71 | 16.70 | 5 |
|  | 40-44 | 13.0 | 145 | 16.7 | 5 | 9.9 | | 35 | 13.30 | 4 |
|  | 45-49 | 7.7 | 86 | 10.0 | 3 | 8.2 | | 29 | 0.00 | 0 |
| **Education** | |  |  |  |  |  | |  |  |  |
|  | Never attended | 10.1 | 113 | 0.0 | 0 | 32.1 | | 113 | 10.0 | 3 |
|  | Primary | 13.3 | 148 | 16.7 | 5 | 37.5 | | 132 | 13.3 | 4 |
|  | Secondary | 50.5 | 563 | 60.0 | 18 | 25.9 | | 91 | 56.7 | 17 |
|  | Higher | 26.0 | 290 | 23.3 | 7 | 4.6 | | 16 | 20.0 | 6 |
| **Marital status** | |  |  |  |  |  | |  |  |  |
|  | Married/cohabiting | 71.4 | 795 | 60.0 | 18 | 65.1 | | 229 | 46.7 | 14 |
|  | Divorced/widowed | 6.7 | 74 | 13.3 | 4 | 8.2 | | 29 | 6.7 | 2 |
|  | Never married | 21.9 | 244 | 26.7 | 8 | 26.7 | | 94 | 46.7 | 14 |
| **Religion of household (Nigeria)** | |  |  |  |  |  | |  |  |  |
|  | Catholic | 17.5 | 194 | 36.7 | 11 | - | | - | - | - |
|  | Other Christian | 52.8 | 586 | 26.7 | 8 |  | |  |  |  |
|  | Muslim | 27.6 | 307 | 36.7 | 11 |  | |  |  |  |
|  | Other | 2.2 | 24 | 0.0 | 0 |  | |  |  |  |
| **Religion of household (Cote d'Ivoire)** | |  |  |  |  |  | |  |  |  |
|  | Catholic | - | - | - | - | 17.3 | | 61 | 40.0 | 12 |
|  | Evangelical |  |  |  |  | 28.1 | | 99 | 23.3 | 7 |
|  | Muslim |  |  |  |  | 21.3 | | 75 | 16.7 | 5 |
|  | Other |  |  |  |  | 21.9 | | 77 | 20.0 | 6 |
|  | No religion |  |  |  |  | 11.4 | | 40 | 0.0 | 0 |
| **Parity** |  |  |  |  |  |  | |  |  |  |
|  | 0 | 24.5 | 273 | 23.3 | 7 | 10.3 | | 36 | 26.7 | 8 |
|  | 1-2 | 28.7 | 319 | 30.0 | 9 | 39.3 | | 138 | 53.3 | 16 |
|  | 3-4 | 27.2 | 303 | 40.0 | 12 | 26.5 | | 93 | 16.7 | 5 |
|  | 5+ | 19.6 | 218 | 6.7 | 2 | 23.4 | | 84 | 3.3 | 1 |
| **Residence** | |  |  |  |  |  | |  |  |  |
|  | Rural | 39.1 | 435 | 30.0 | 9 | 36.9 | | 130 | 3.3 | 1 |
|  | Urban | 61.0 | 679 | 70.0 | 21 | 63.1 | | 222 | 96.7 | 29 |
| **State (Nigeria)** | |  |  |  |  |  | |  |  |  |
|  | Anambra | 17.2 | 192 | 50.0 | 15 | - | | - | - | - |
|  | Kaduna | 18.5 | 206 | 50.0 | 15 |  | |  |  |  |
|  | Lagos | 21.2 | 236 | - | - |  | |  |  |  |
|  | Nasarawa | 14.5 | 161 |  |  |  | |  |  |  |
|  | Rivers | 23.0 | 256 |  |  |  | |  |  |  |
|  | Taraba | 5.7 | 63 |  |  |  | |  |  |  |
| **Ethnicity (Nigeria)** | |  |  |  |  |  | |  |  |  |
|  | Hausa | 12.5 | 139 | 20.0 | 6 | - | | - | - | - |
|  | Igbo | 25.8 | 286 | 50.0 | 15 |  | |  |  |  |
|  | Yoruba | 13.0 | 145 | 3.3 | 1 |  | |  |  |  |
|  | Other | 48.7 | 543 | 26.7 | 8 |  | |  |  |  |
| **Ethnicity (Cote d'Ivoire)** | |  |  |  |  |  | |  |  |  |
|  | Akan | - | - | - | - | 53.4 | | 188 | 40.0 | 12 |
|  | Mande |  |  |  |  | 15.1 | | 53 | 23.3 | 7 |
|  | Gur |  |  |  |  | 9.7 | | 34 | 10.0 | 3 |
|  | Other Ivoirian |  |  |  |  | 13.9 | | 49 | 13.3 | 4 |
|  | Other non-Ivoirian |  |  |  |  | 8.0 | | 28 | 13.3 | 4 |
| *Note: Qualitative sample characteristics are presented from the baseline survey while quantitative sample characteristics are presented from the follow-up survey.* | | | | | | | | | | |
