# Supplementary material for: A mixed-methods study exploring women’s perceptions of terminology surrounding fertility and menstrual regulation in Côte d’Ivoire and Nigeria
Source: Reprod Health. 2021 Dec 20;18:251. doi: 10.1186/s12978-021-01306-5 (PMC8686364; doi:10.1186/s12978-021-01306-5)
Supplement: Supplementary file 2 — Additional file 2: Table S2. Percentage that consider pregnancy removal different than period regulation, by background characteristics. [file 12978_2021_1306_MOESM2_ESM.docx]

| **Table S2. Percentage that consider pregnancy removal different than period regulation, by background characteristics (% weighted, N unweighted)** | | | | | | | |
| --- | --- | --- | --- | --- | --- | --- | --- |
|  |  | **Nigeria (n=1114)** | | | **Cote d'Ivoire (n= 352)** | | |
|  |  | % | N | P-value | % | N | P-value |
| Overall | | 70.8 | 789 |  | 69.9 | 246 |  |
| ***Demographic measures*** | |  |  |  |  |  |  |
| **Age** | |  |  | 0.99 |  |  | 0.81 |
|  | 15-19 | 71.4 | 40 |  | 65.0 | 13 |  |
|  | 20-24 | 68.9 | 115 |  | 72.9 | 43 |  |
|  | 25-29 | 70.0 | 166 |  | 69.4 | 50 |  |
|  | 30-34 | 70.4 | 162 |  | 71.2 | 47 |  |
|  | 35-39 | 71.5 | 138 |  | 73.2 | 52 |  |
|  | 40-44 | 73.1 | 106 |  | 68.6 | 24 |  |
|  | 45-49 | 72.1 | 62 |  | 58.6 | 17 |  |
| **Education** | |  |  | 0.62 |  |  | 0.77 |
|  | Never | 69.0 | 78 |  | 67.3 | 76 |  |
|  | Primary | 75.7 | 112 |  | 72.7 | 96 |  |
|  | Secondary | 70.0 | 394 |  | 68.1 | 62 |  |
|  | Higher | 70.7 | 205 |  | 75.0 | 12 |  |
| **Marital status** | |  |  | 0.60 |  |  | 0.72 |
|  | Married/cohabiting | 70.8 | 563 |  | 71.6 | 164 |  |
|  | Divorced or separated/widowed | 75.7 | 56 |  | 69.0 | 20 |  |
|  | Never married | 69.7 | 170 |  | 66.0 | 62 |  |
| **Religion of household (Nigeria)** | |  |  | 0.12 |  |  |  |
|  | Catholic | 70.6 | 137 |  | - | - |  |
|  | Other Christian | 69.8 | 409 |  |  |  |  |
|  | Muslim | 71.3 | 219 |  |  |  |  |
|  | Other | 95.8 | 23 |  |  |  |  |
| **Religion of household (Cote d'Ivoire)** | |  |  |  |  |  | 0.47 |
|  | Catholic | - | - |  | 75.8 | 36 |  |
|  | Evangelical |  |  |  | 72.0 | 75 |  |
|  | Muslim |  |  |  | 59.0 | 54 |  |
|  | Other |  |  |  | 68.8 | 53 |  |
|  | No religion |  |  |  | 70.0 | 27 |  |
| **Parity** | |  |  | 0.83 |  |  | 0.34 |
|  | 0 | 70.3 | 192 |  | 77.8 | 28 |  |
|  | 1-2 | 69.3 | 221 |  | 70.3 | 97 |  |
|  | 3-4 | 71.3 | 216 |  | 73.1 | 68 |  |
|  | 5+ | 72.9 | 159 |  | 61.9 | 52 |  |
| **Residence** | |  |  | 0.09 |  |  | 0.77 |
|  | Rural | 74.3 | 323 |  | 71.5 | 93 |  |
|  | Urban | 68.6 | 466 |  | 68.9 | 153 |  |
| **State (Nigeria)** | |  |  | 0.28 |  |  |  |
|  | Anambra | 68.8 | 132 |  | - | - |  |
|  | Kaduna | 71.4 | 147 |  |  |  |  |
|  | Lagos | 75.9 | 179 |  |  |  |  |
|  | Nasarawa | 64.0 | 103 |  |  |  |  |
|  | Rivers | 69.5 | 178 |  |  |  |  |
|  | Taraba | 79.4 | 50 |  |  |  |  |
| **Ethnicity (Nigeria)** | |  |  | 0.82 |  |  |  |
|  | Hausa | 67.6 | 94 |  | - | - |  |
|  | Igbo | 70.4 | 202 |  |  |  |  |
|  | Yoruba | 73.8 | 107 |  |  |  |  |
|  | Other | 71.1 | 386 |  |  |  |  |
| **Ethnicity (Cote d'Ivoire)** | |  |  |  |  |  | 0.14 |
|  | Akan | - | - |  | 77.1 | 145 |  |
|  | Mande |  |  |  | 52.8 | 28 |  |
|  | Gur |  |  |  | 58.8 | 20 |  |
|  | Other Ivoirian |  |  |  | 67.4 | 33 |  |
|  | Other non-Ivoirian |  |  |  | 71.4 | 20 |  |
| ***Abortion-specific measures*** | |  |  |  |  |  |  |
| **Certainty about pregnancy that was terminated** | | |  | **<0.001** |  |  | **0.02** |
|  | Very certain | 66.5 | 481 |  | 65.7 | 163 |  |
|  | Certain | 74.3 | 153 |  | 62.2 | 23 |  |
|  | Not certain | 85.5 | 147 |  | 89.4 | 59 |  |
| **Took pregnancy test** | |  |  |  |  |  |  |
|  | Yes | 67.7 | 401 | 0.10 | 73.8 | 118 | 0.20 |
|  | No | 74.2 | 385 |  | 65.9 | 127 |  |
| **Abortion method used (first/only method)** | | |  | 0.08 |  |  | 0.66 |
|  | Surgery | 65.4 | 198 |  | 73.6 | 81 |  |
|  | Medication abortion | 67.7 | 42 |  | 76.9 | 10 |  |
|  | Other pills | 70.8 | 211 |  | 68.2 | 45 |  |
|  | Other (e.g. traditional) | 74.5 | 327 |  | 67.3 | 109 |  |
| **Reported period regulation vs. pregnancy removal** | | | | **0.01** |  |  | 0.80 |
|  | Period regulation | 75.8 | 276 |  | 71.8 | 79 |  |
|  | Pregnancy removal | 68.4 | 513 |  | 69.0 | 167 |  |
|  |  | |  |  |  |  |  |
