# Supplementary material for: A mixed-methods study exploring women’s perceptions of terminology surrounding fertility and menstrual regulation in Côte d’Ivoire and Nigeria
Source: Reprod Health. 2021 Dec 20;18:251. doi: 10.1186/s12978-021-01306-5 (PMC8686364; doi:10.1186/s12978-021-01306-5)
Supplement: Supplementary file 3 — Additional file 3: Table S3. Perceptions of scenarios. [file 12978_2021_1306_MOESM3_ESM.docx]

| **Table S3. Perceptions of scenarios (% weighted, N unweighted)** | | | | |  |
| --- | --- | --- | --- | --- | --- |
|  |  | Agrees period regulation | | Agrees pregnancy removal |  |
| **Nigeria (n=1114)*** | | % | | % | P-value |
| Taking a pill within a couple days after unprotected sex | | 69.1 | | 16.5 | **<0.001** |
| Taking pills after missing 1-2 periods without pregnancy confirmation | | 56.5 | | 45.4 | **<0.001** |
| Having a procedure after missing 1-2 periods without pregnancy confirmation | | 26.1 | | 54.5 | **<0.001** |
| Taking pills when a woman is sure she is early in a pregnancy | | 18.5 | | 82.9 | **<0.001** |
| Having surgery when a woman is sure she is early in a pregnancy | | 13.4 | | 83.8 | **<0.001** |
| Taking pills when the pregnancy has been confirmed | | 14.7 | | 85.5 | **<0.001** |
| Having a surgery when the pregnancy has been confirmed | | 12.0 | | 79.7 | **<0.001** |
| Taking pills after a miscarriage | | 26.9 | | 7.4 | **<0.001** |
| Having a surgery after a miscarriage | | 21.8 | | 7.5 | **<0.001** |
| **Cote d'Ivoire (n=352)** | | % | | % | P-value |
| Taking a pill within a couple days after unprotected sex | | 54.0 | | 25.3 | **<0.001** |
| Taking pills after missing 1-2 periods without pregnancy confirmation | | 65.1 | | 33.5 | **<0.001** |
| Having a procedure after missing 1-2 periods without pregnancy confirmation | | 58.0 | | 32.1 | **<0.001** |
| Taking pills when a woman is sure she is early in a pregnancy | | 25.6 | | 77.6 | **<0.001** |
| Having surgery when a woman is sure she is early in a pregnancy | | 22.7 | | 74.2 | **<0.001** |
| Taking pills when the pregnancy has been confirmed | | 22.2 | | 83.0 | **<0.001** |
| Having a surgery when the pregnancy has been confirmed | | 16.8 | | 67.1 | **<0.001** |
| Taking pills after a miscarriage | | 21.3 | | 2.0 | **<0.001** |
| Having a surgery after a miscarriage | | 21.0 | | 0.6 | **<0.001** |
|  |  |  |  | |  |
|  |  |  |  | |  |
|  | ** In Nigeria, 1,040 women completed the period regulation questions and 1,114 completed the pregnancy removal questions* | | | |  |
|  |  |  |  | |  |
