# Supplementary material for: A mixed-methods study exploring women’s perceptions of terminology surrounding fertility and menstrual regulation in Côte d’Ivoire and Nigeria
Source: Reprod Health. 2021 Dec 20;18:251. doi: 10.1186/s12978-021-01306-5 (PMC8686364; doi:10.1186/s12978-021-01306-5)
Supplement: Supplementary file 4 — Additional file 4: Table S4. Perceptions of scenarios according to whether respondent reported menstrual regulation or pregnancy removal. [file 12978_2021_1306_MOESM4_ESM.docx]

| **Table S4. Perceptions of scenarios according to whether respondent reported menstrual regulation or pregnancy removal (% weighted, N unweighted)** | | | | | | | | | | | |
| --- | --- | --- | --- | --- | --- | --- | --- | --- | --- | --- | --- |
|  | **Agrees period regulation** | | | | | **Agrees pregnancy removal** | | | | | |
|  | Reported period regulation | Reported pregnancy removal | |  | | Reported period regulation | | Reported pregnancy removal | |  | |
| **Nigeria*** | % (N=351) | % (N=689) | | P-value | | % (N=364) | | % (N=750) | | P-value | |
| Taking a pill within a couple days after unprotected sex | 69.8 | 68.8 | | 0.74 | | 14.3 | | 17.6 | | 0.16 | |
| Taking pills after missing 1-2 periods without pregnancy confirmation | 59.8 | 54.9 | | 0.13 | | 40.1 | | 48.0 | | **0.01** | |
| Having a procedure after missing 1-2 periods without pregnancy confirmation | 23.1 | 27.6 | | 0.12 | | 48.9 | | 57.2 | | **0.01** | |
| Taking pills when a woman is sure she is early in a pregnancy | 14.3 | 20.6 | | **0.01** | | 82.4 | | 83.1 | | 0.79 | |
| Having surgery when a woman is sure she is early in a pregnancy | 9.4 | 15.4 | | **0.01** | | 80.8 | | 85.3 | | **0.05** | |
| Taking pills when the pregnancy has been confirmed | 13.7 | 15.2 | | 0.50 | | 86.3 | | 85.1 | | 0.60 | |
| Having a surgery when the pregnancy has been confirmed | 9.1 | 13.5 | | **0.04** | | 78.0 | | 80.5 | | 0.33 | |
| Taking pills after a miscarriage | 26.5 | 27.1 | | 0.82 | | 6.9 | | 7.6 | | 0.66 | |
| Having a surgery after a miscarriage | 20.8 | 22.4 | | 0.57 | | 6.3 | | 8.1 | | 0.28 | |
| **Cote d'Ivoire** | % (N=110) | % (N=242) | | P-value | | % (N=110) | | % (N=242) | | P-value | |
| Taking a pill within a couple days after unprotected sex | 41.8 | 59.5 | | **0.002** | | 15.5 | | 29.8 | | **0.004** | |
| Taking pills after missing 1-2 periods without pregnancy confirmation | 60.9 | 66.9 | | 0.27 | | 20.0 | | 39.7 | | **<0.001** | |
| Having a procedure after missing 1-2 periods without pregnancy confirmation | 52.7 | 60.3 | | 0.18 | | 20.0 | | 37.6 | | **0.001** | |
| Taking pills when a woman is sure she is early in a pregnancy | 12.7 | 31.4 | | **<0.001** | | 68.2 | | 81.8 | | **0.004** | |
| Having surgery when a woman is sure she is early in a pregnancy | 12.7 | 27.3 | | **0.003** | | 59.1 | | 81.0 | | **<0.001** | |
| Taking pills when the pregnancy has been confirmed | 14.6 | 25.6 | | **0.02** | | 75.5 | | 86.4 | | **0.01** | |
| Having a surgery when the pregnancy has been confirmed | 10.9 | 19.4 | | **0.05** | | 55.5 | | 72.3 | | **0.002** | |
| Taking pills after a miscarriage | 19.1 | 22.3 | | 0.50 | | 0.9 | | 2.5 | | 0.33 | |
| Having a surgery after a miscarriage | 19.1 | 21.9 | | 0.55 | | 0.0 | | 0.8 | | 0.34 | |
|  |  | |  | |  | |  | |  | |  |
|  |  | |  | |  | |  | |  | |  |
| ** In Nigeria, 1,040 women completed the period regulation questions and 1,114 completed the pregnancy removal questions* | | | | | | | | |  | |  |
|  | | | | | | | | |  | |  |
