# Supplementary material for: A mixed-methods study exploring women’s perceptions of terminology surrounding fertility and menstrual regulation in Côte d’Ivoire and Nigeria
Source: Reprod Health. 2021 Dec 20;18:251. doi: 10.1186/s12978-021-01306-5 (PMC8686364; doi:10.1186/s12978-021-01306-5)
Supplement: Supplementary file 5 — Additional file 5: Table S5. Perceptions of scenarios according to whether respondent was certain or not about being pregnant for the pregnancy that was terminated. [file 12978_2021_1306_MOESM5_ESM.docx]

| **Table S5. Perceptions of scenarios according to whether respondent was certain or not about being pregnant for the pregnancy that was terminated**  **(% weighted, N unweighted)** | | | | | | | | | | | |
| --- | --- | --- | --- | --- | --- | --- | --- | --- | --- | --- | --- |
|  | **Agrees period regulation** | | | | | | **Agrees pregnancy removal** | | | | |
|  | Certain | Not certain | |  | | | Certain | | Not certain | |  |
| **Nigeria*** | % (N=875) | % (N=157) | | P-value | | | % (N=929) | | % (N=172) | | P-value |
| Taking a pill within a couple days after unprotected sex | 69.7 | 66.9 | | 0.48 | | | 16.0 | | 18.6 | | 0.40 |
| Taking pills after missing 1-2 periods without pregnancy confirmation | 56.1 | 59.2 | | 0.47 | | | 46.4 | | 37.8 | | **0.04** |
| Having a procedure after missing 1-2 periods without pregnancy confirmation | 27.1 | 20.4 | | 0.08 | | | 54.8 | | 50.6 | | 0.31 |
| Taking pills when a woman is sure she is early in a pregnancy | 19.5 | 12.1 | | **0.03** | | | 83.0 | | 81.4 | | 0.61 |
| Have a surgery when a woman is sure she is early in a pregnancy | 14.5 | 6.4 | | **0.01** | | | 83.4 | | 86.6 | | 0.29 |
| Taking pills when the pregnancy has been confirmed | 15.2 | 10.8 | | 0.15 | | | 85.5 | | 85.5 | | 1.00 |
| Having a surgery when the pregnancy has been confirmed | 12.7 | 7.0 | | **0.04** | | | 79.9 | | 79.7 | | 0.95 |
| Taking pills after a miscarriage | 27.3 | 24.2 | | 0.42 | | | 7.3 | | 7.6 | | 0.91 |
| Having a surgery after a miscarriage | 21.8 | 21.0 | | 0.82 | | | 7.2 | | 9.9 | | 0.23 |
| **Cote d'Ivoire** | % (N=285) | % (N=66) | | P-value | | | % (N=285) | | % (N=66) | | P-value |
| Taking a pill within a couple days after unprotected sex | 56.5 | 42.4 | | **0.04** | | | 25.6 | | 22.7 | | 0.63 |
| Taking pills after missing 1-2 periods without pregnancy confirmation | 67.0 | 56.1 | | 0.09 | | | 34.4 | | 28.8 | | 0.39 |
| Having a procedure after missing 1-2 periods without pregnancy confirmation | 57.5 | 59.1 | | 0.82 | | | 34.4 | | 21.2 | | **0.04** |
| Taking pills when a woman is sure she is early in a pregnancy | 28.4 | 13.6 | | **0.01** | | | 78.3 | | 74.2 | | 0.48 |
| Have a surgery when a woman is sure she is early in a pregnancy | 25.6 | 10.6 | | **0.01** | | | 76.5 | | 63.6 | | **0.03** |
| Taking pills when the pregnancy has been confirmed | 24.6 | 12.1 | | **0.03** | | | 83.9 | | 78.8 | | 0.33 |
| Having a surgery when the pregnancy has been confirmed | 19.3 | 6.1 | | **0.01** | | | 69.1 | | 59.1 | | 0.12 |
| Taking pills after a miscarriage | 20.7 | 24.2 | | 0.53 | | | 1.4 | | 4.6 | | 0.10 |
| Having a surgery after a miscarriage | 20.4 | 24.2 | | 0.49 | | | 0.35 | | 1.5 | | 0.26 |
|  |  | |  | |  |  | |  | |  | |
|  |  | |  | |  |  | |  | |  | |
| ** In Nigeria, 1,040 women completed the period regulation questions and 1,114 completed the pregnancy removal questions* | | | | | | | |  | |  | |
|  |  | |  | |  |  | |  | |  | |
