# Supplementary figures and images for: A mixed-methods study exploring women’s perceptions of terminology surrounding fertility and menstrual regulation in Côte d’Ivoire and Nigeria
Source: Reprod Health. 2021 Dec 20;18:251. doi: 10.1186/s12978-021-01306-5 (PMC8686364; doi:10.1186/s12978-021-01306-5)

***Figure 1a. Côte d’Ivoire word cloud***


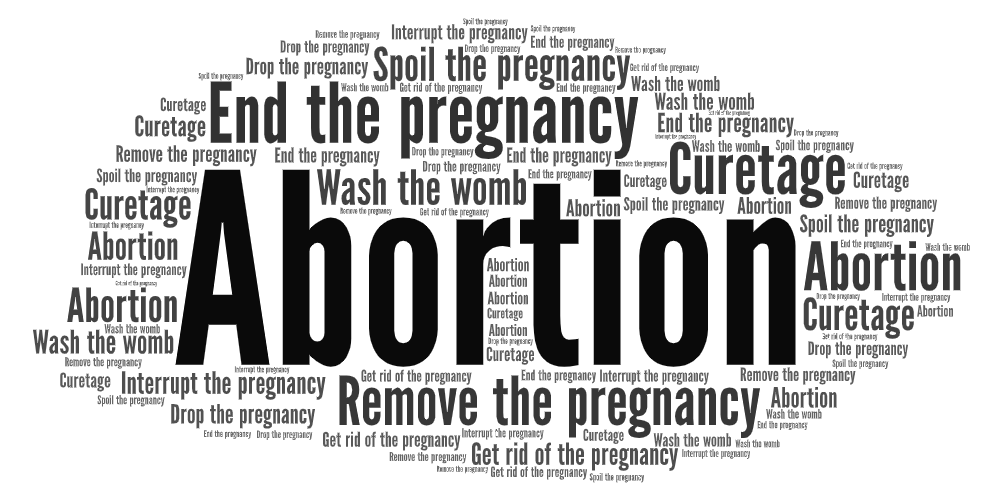


***Figure 1b. Nigeria word cloud***

***
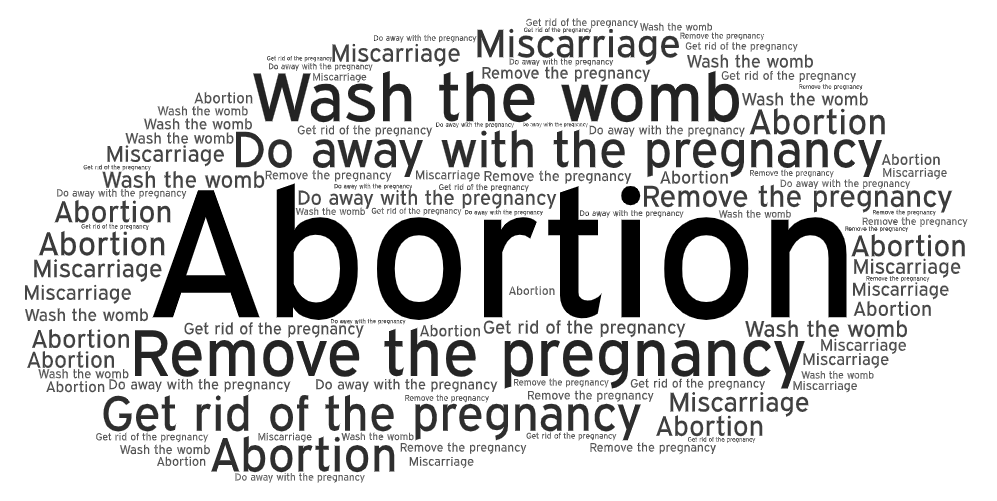
***

Supplement: Supplementary file 6 — Additional file 6: Fig. S1. Word clouds highlighting terminology used by qualitative interview participants in Cote d’Ivoire and Nigeria. [file 12978_2021_1306_MOESM6_ESM.docx]
